# Primary sclerosing cholangitis with PLKR and UGT1A1 mutation manifested as recurrent bile duct stones: A case report

**DOI:** 10.1097/MD.0000000000041192

**Published:** 2025-01-03

**Authors:** Yijun Zhou, Wei Shen, Yusheng Cui, Yunjiang Li, Hong Liu, Jianfeng Bao, Qiaofei Jin

**Affiliations:** a Department of Hepatology, Hangzhou Xixi Hospital, Hangzhou, Zhejiang Province, China; b Department of Radiology, Hangzhou Xixi Hospital, Hangzhou, Zhejiang Province, China; c Department of Pathology, Hangzhou Xixi Hospital, Hangzhou, Zhejiang Province, China.

**Keywords:** bile duct stones, case report, primary sclerosing cholangitis, vanishing bile duct syndrome, whole exome sequencing

## Abstract

**Rationale:**

Primary sclerosing cholangitis (PSC) is characterized by idiopathic intra- and extrahepatic bile duct inflammation and biliary fibrotic changes. Recurrent bile duct stones due to PLKR and UGT1A1 mutation is an extremely rare complications of PSC.

**Patient concerns:**

A 26-year-old male patient complains a history of recurrent yellow skin and urine for over a year.

**Diagnoses:**

Following dynamic magnetic resonance cholangiopancreatography imaging, colonoscopic manifestation, liver biopsy and whole exome sequencing, the patient was finally diagnosed with PSC - ulcerative colitis with PLKR and UGT1A1 mutation.

**Interventions:**

Following resolution of the obstruction, a long-term regimen of 1000 mg/d ursodeoxycholic acid in combination with 10 mg/d obeticholic acid to improve cholestasis, 8 g/d colestyramine to facilitate adsorption of excess bile acids and 1.2 g/d rifaximin to prevent biliary tract infection were prescribed.

**Outcomes:**

The patient’s liver biochemical parameters have improved significantly. His condition is stable and has not undergone liver transplantation at this time.

**Lessons:**

Close and dynamic detection of the patient’s biliary ductal lesions play an important role in the diagnosis of PSC. In the event of relatively rare biliary complications, attention should be paid to the presence of gene mutation.

## 1. Introduction

Primary sclerosing cholangitis (PSC) is a subtype of autoimmune liver diseases, which is characterized by idiopathic intra- and extrahepatic bile duct inflammation and biliary fibrotic changes. These lead to the formation of multifocal bile duct strictures and chronic cholestasis.^[1]^ Early diagnosis is challenging due to the fact that the classical imaging of the bile ducts does not occur in the early stage.^[2]^ Furthermore, approximately 30% of PSC patients have a combination of inflammatory bowel disease (IBD).^[3]^ It is estimated that over 80% of PSC patients concomitant with IBD are PSC-ulcerative colitis (UC).^[4]^ Patients with PSC-UC are more likely to present with pancolitis, backwash ileitis, and rectal sparing. In comparison with UC alone, patients with PSC-UC are frequently asymptomatic or display only minimal symptoms. The endoscopic presentation of the colonic mucosa may be normal, but biopsy often reveals microscopic colitis.^[5]^ Patients with PSC-UC are at a significantly elevated risk of developing colorectal and hepatobiliary system carcinoma in comparison to patients with UC alone.^[6]^ Implementing regular colonoscopy screening proves beneficial in improving clinical outcomes in patients with PSC-UC.^[7]^ Furthermore, the common complications of PSC are bile duct stricture, recurrent biliary infections and even cholangiocarcinoma.^[8]^ However, recurrent bile duct stones is extremely rare. This case report a PSC patient with recurrent bile duct stones and heterozygous mutations in PLKR and UGT1A1, together with a detailed account of the diagnosis and treatment.

## 2. Case presentation

**Figure 1.**
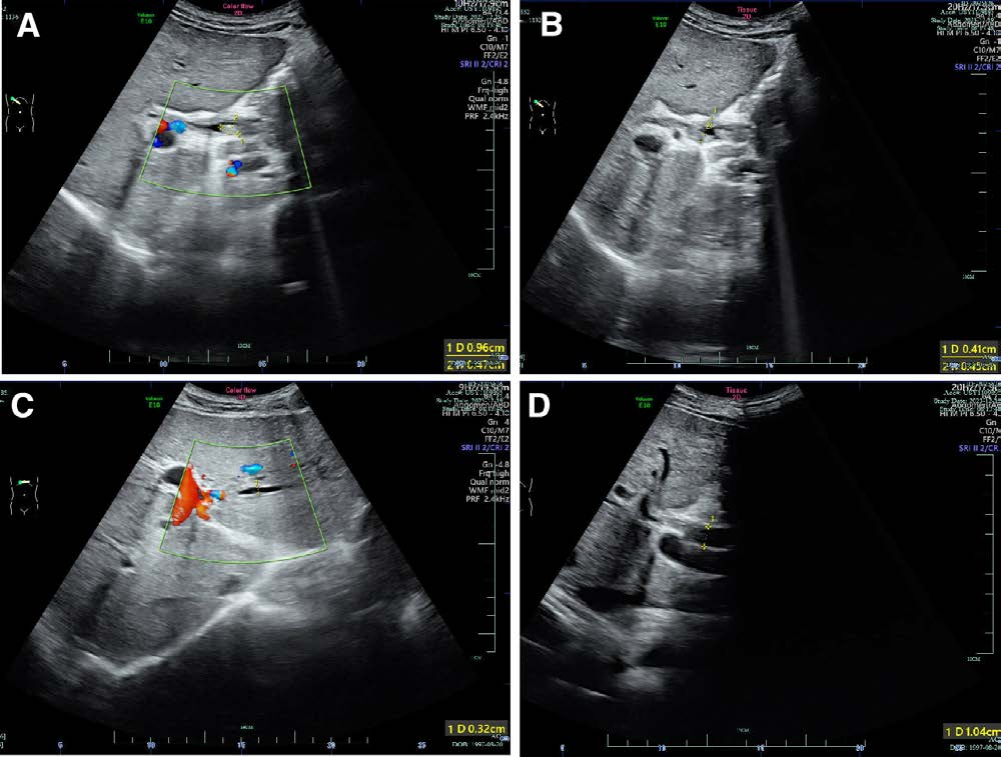
Ultra sound manifestation. The presence of multiple stones in the common bile duct (A, B), with mild dilatation of the intrahepatic (C) and common bile duct (D).

A 26-year-old male patient was admitted to our hospital with a history of recurrent yellow skin and urine for over a year. On June 1, 2022, the patient presented with yellow skin and urine, without any obvious triggers. An ultrasound examination revealed the presence of multiple stones in the common and intrahepatic bile duct, with mild dilatation of the intrahepatic (diameter: 0.32 cm, reference: ≤ 0.2 cm) and common bile duct (diameter: 1.04 cm, reference: 0.6–0.8 cm; Fig. 1). These findings supported the initial impression of obstructive jaundice. On June 15, 2022, the patient underwent laparoscopic choledocholithotomy, cholecystectomy and choledochoscopic lithotomy. However, there was no improvement in liver function nor symptoms of jaundice after surgery over the past year. In order to seek further medical assistance, the patient was admitted to our hospital with icterus of unknown origin on August 12, 2023. The patient denied a history of smoking, alcohol abuse, and long-term use of liver-damaging medication, including herbal and dietary supplements.

The physical examination revealed the presence of generalized yellowing of the skin and sclera, as well as a palpable spleen with 2 fingers below the rib cage. Additionally, the subject’s body mass index was recorded at 17.4 kg/m².

The results of vital laboratory examinations were presented in Table 1. Additionally, antinuclear antibody (ANA) 1:100, p-antineutrophil cytoplasmic antibodies (p-ANCA) positive, autoimmune liver diseases antibody profile were all negative; hepatitis A to E, Epstein–Barr virus, cytomegalovirus, coxsackie virus, herpes zoster virus, treponema pallidum and human immunodeficiency virus were all negative.

**Table 1:** Vital laboratory result.

		Value	Unit	Reference value	
				Min	Max
Blood routine test	Hemoglobin	113 ↓	g/L	115	150
	Platelet	118 ↓	10^9^/L	125	350
	Reticulocyte	0.065	10^12^/L	0.024	0.085
Liver function	Total bilirubin	166.47 ↑	μmol/L	0	23
	Direct bilirubin	122.01 ↑	μmol/L	0	6.84
	Albumin	33.9 ↓	g/L	40	55
	Globulin	47.5 ↑	g/L	20	40
	ALT	168 ↑	U/L	9	50
	AST	165 ↑	U/L	15	40
	ALP	544 ↑	U/L	30	120
	GGT	336 ↑	U/L	10	60
Immunoglobulin	IgG	23.59 ↑	g/L	8.6	17.4
	IgG4	2.19 ↑	g/L	0.36	2.09
	Prothrombin time	12.7 ↑	s	9.7	12.6
	Ceruloplasmin	0.26	g/L	0.2	0.6
	CA 199	181 ↑	KU/L	0	35

ALP = alkaline phosphatase, ALT = alanine aminotransferase, AST = aspartate aminotransferase, CA = carbohydrate antigen, GGT = gamma-glutamyl transpeptidase, Ig = immunoglobulin.

**Figure 2.**
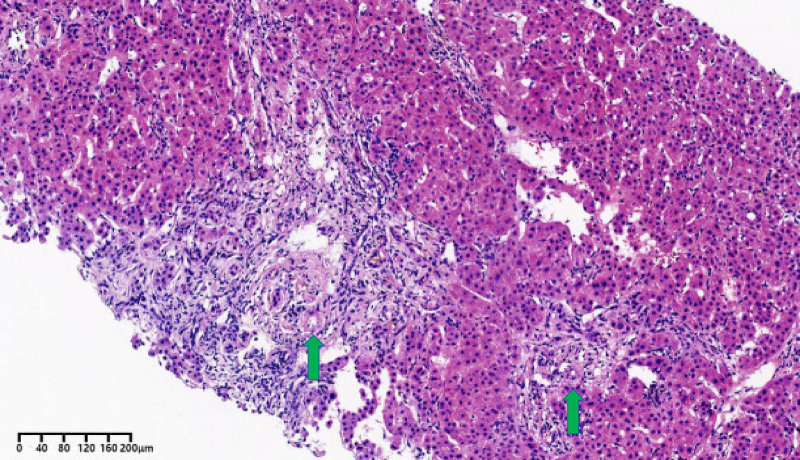
Histological characteristics of liver biopsy. Interlobular bile ducts, accompanied by interlobular arteries (green arrow), were absent in most portal tracts.

Magnetic resonance cholangiopancreatography (MRCP) demonstrated the existence of dilated left intrahepatic and common bile duct, in the absence of a gallbladder, splenomegaly and multiple enlarged lymph nodes in the hilar region. The histological examination of the liver revealed that the structure of the hepatic lobules and the capillary bile duct network were not clearly discernible. Some capillary bile ducts displayed dilatation, accompanied by the formation of bile thrombus. There was also bridging fibrosis from the portal tracts to the central vein. Disappearance of bile ducts was observed in 10 of the 12 portal tracts (Fig. 2). This phenomenon is most commonly attributed to vanishing bile duct syndrome (VBDS).^[9]^ The simplified Scheuer score^[10]^ indicated a grade 2 and stage 3 to 4. Whole exome sequencing revealed the presence of a heterozygous mutation in PKLR (NM_000298.5:c.119G > A) and UGT1A1 (NM_000463.2:c.–3275T > G).

In light of the aforementioned evidence, a revised diagnosis was rendered as VBDS with PLKR and UGT1A1 mutation, cholestatic cirrhosis. A dosage of 1000 mg/d ursodeoxycholic acid (UDCA) and 8 mg/d methylprednisolone was administered in order to improve cholestasis and hepatic inflammation.

**Figure 3.**
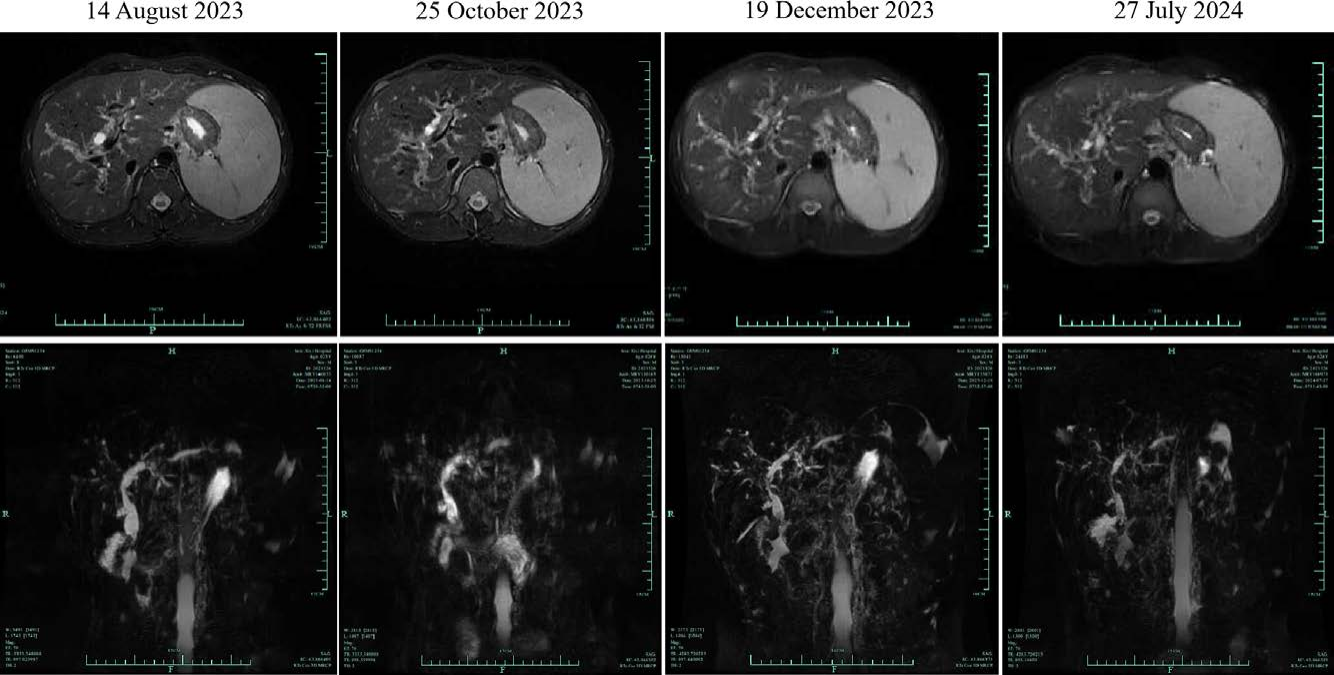
Dynamic changes of biliary duct lesion by MRCP. MRCP = magnetic resonance cholangiopancreatography.

**Figure 4.**
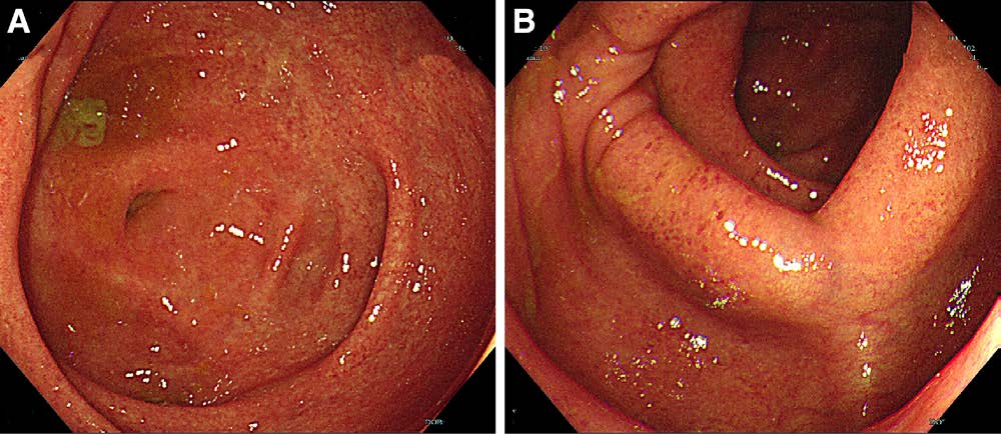
Colonoscopy manifestation showed the presence of ulcerative colitis (UC) involving the terminal ileum (A) and right hemicolon (B). UC = ulcerative colitis.

Over time, a contrast-enhanced magnetic resonance imaging (MRI) scan performed on September 10, 2023 revealed the presence of stones in the common bile duct, despite the absence of fever, abdominal pain, or other complaints. The patient refused to have the stones removed from the common bile duct. On October 25, 2023, an MRCP examination showed dynamic changes. The biliary system showed banded stricture and a pruned tree appearance, which was in consistent with the imaging appearance of sclerosing cholangitis (Fig. 3). Further colonoscopy was performed with adequate bowel preparation, adhering to guidelines for patients with suspected mucosal damage,^[11]^ which revealed the presence of UC involving the terminal ileum and right hemicolon (Fig. 4). This finding was consistent with the colonoscopic manifestations of PSC-UC.^[12]^ Moreover, PSC-UC is a unique subtype of UC, which makes it difficult to apply Montreal classification criteria to describe the disease’s topography. Consequently, the diagnosis was revised to PSC-UC with PLKR and UGT1A1 mutations, choledocholithiasis.

On November 25, 2023, transduodenoscopic retrograde cholangiopancreatography (ERCP) and endoscopic sphincterotomy (EST) were performed. A 4 mm × 5 mm black irregular stone and a large number of sedimentary stones were successfully removed using endoscopic duodenal papillary sphincter dilatation (EPBD). Following resolution of the obstruction, a long-term regimen of 1000 mg/d UDCA in combination with 10 mg/d obeticholic acid (OCA) was prescribed to improve cholestasis. In addition, 8 g/d colestyramine was prescribed to facilitate the adsorption of excess bile acids, while 1.2 g/d rifaximin was administered to prevent biliary tract infection. Furthermore, given the fact that the patient’s UC was limited to the terminal ileum and right hemicolon, there was a paucity of mucopurulent and bloody stools, as well as diarrhea. Mesalazine tablets (2 g per day) was medicated with the objective of improving colonic inflammation. On July 30, 2024, the patent’s liver function was as follows TB/DB 33/25.9 μmol/L, alkaline phosphatase (ALP)/GGT 183/112 U/L, IgG 17.19 g/L. The patient’s condition remained stable.

## 3. Discussion

The following 6 features were shown in our case: Young man with recurrent bile duct stones as the most prominent manifestation; A mixed pattern of liver damage with cholestasis predominant, and immune indicators showed an ANA titer of 1:100 with p-ANCA positivity and elevated levels of IgG and IgG4; Pathological examination showed pure severe cholestasis and VBDS, with no obvious interface inflammation; MRCP showed dynamic changes in the bile ducts and typical sclerosing cholangitis appeared in the late stage, accompanied by portal hypertension and splenomegaly; Backwash colitis involving the distal ileum and right hemicolon was present, which was in consistent with the colonoscopic manifestations of PSC-UC; PKLR and UGT1A1 mutations were detected. Thus, there were 3 major breakthroughs in the diagnosis process.

Firstly, VBDS was the prominent pathological manifestation. Lv et al^[13]^ revealed that the etiological components of VBDS, in descending order, were primary biliary cholangitis, drug-induced liver injury, adult idiopathic bile duct loss, PSC, Alagille syndrome and progressive familial intrahepatic cholestasis (PFIC). For the extrahepatic bile duct changes on MRCP, the focus of the patient was on PSC and PFIC. In terms of pure intrahepatic cholestasis with recurrent gallbladder stones, PFIC-3 due to ABCB4 mutation should be considered until whole exome sequencing are available. ABCB4 mainly encodes multidrug resistance protein 3, which regulates the secretion of phospholipids, which are used to emulsify bile salts and cholesterol.^[14]^ When the ABCB4 gene is mutated, phospholipids cannot be transported into the bile duct, which leads to an increase in the ratio of cholesterol and bile salts in the bile, resulting in bile duct damage, cholestasis, recurrent formation of gallbladder and bile duct stones and even biliary liver cirrhosis in severe cases.^[15]^ PFIC-3 support the patient’s clinical manifestations such as recurrent bile duct stones and disappearance of bile ducts, while the result of WSE does not support the diagnosis.

Secondly, the presence of heterozygous mutations in both PKLR and UGT1A1 should be noted. PKLR mutation is closely associated with pyruvate kinase (PK) deficiency, resulting in insufficient ATP production and ultimately leading to hemolysis.^[16]^ ATP deficiency causes sodium ions to accumulate in erythrocytes, leading to spherical shape of erythrocytes and subsequent destruction as they pass through the spleen, resulting in hemolytic anemia, which are typically only developed by pure or compound heterozygotes.^[17]^ Cholelithiasis is the most common complication of PK deficiency.^[18]^ Furthermore, heterozygous mutations in UGT1A1 result in reduced glucuronosyltransferase activity, leading to physiological icterus.^[19]^ Therefore, the combined mutation of PKLR and UGT1A1 results in long-term disorders of bilirubin metabolism, causing recurrent formation of bile duct stones. However, mutations in PKLR and UGT1A1 make it challenging to explain the dynamic changes in the bile duct.

Thirdly, there are dynamic changes in the extrahepatic bile ducts over time, eventually progressing to sclerosing cholangitis. Palak Trivedi and colleagues^[20]^ revealed that the course of PSC is not static, but can change with time. The progression from atypical PSC features to predominantly PSC features is not only seen in children, but also in adolescents and young adults. Therefore, a long-term follow-up cohort should be established to determine the diagnosis of PSC. In the case of this patient, a long history of cholestasis with imaging typical of PSC over time, together with UC, is entirely consistent with a diagnosis of PSC. However, the most common complications of PSC are recurrent biliary tract infections and biliary strictures, whereas cholelithiasis is relatively rare.

Based on the 3 clues mentioned above, the final diagnosis of the patient was PSC - UC with PLKR and UGT1A1 mutation. Up until now, PKLR and UGT1A1 double mutations have been reported only in a 15-year-old child with PK deficiency combined with Crigler–Najjar syndrome type II.^[21]^ No cases were reported in PSC patient.

Moreover, there are no effective therapeutic agents for PSC. Small doses of UDCA (10–15 mg/kg/d) improved liver biochemistry but not long-term clinical endpoints such as liver transplantation and death. Large doses of UDCA not only had no benefits but also increased the risk of death, liver transplantation and the incidence of serious adverse events.^[22]^ However, based on the data available, no specific recommendations can be made for the routine dose of UDCA. OCA is a selective Farnesoid X Receptor agonist that effectively inhibits bile acid synthesis. A phase II randomized, double-blind, placebo-controlled clinical trial was conducted to investigate the efficacy of obeticholic acid in adult patients with non-cirrhotic or compensated PSC and elevated ALP levels. The results demonstrated that 5 to 10 mg/d of obeticholic acid can significantly reduce ALP levels over a 24-week treatment period.^[23]^

In conclusion, this case represents the inaugural report of a PSC patient with a double heterozygous mutation of PKLR and UGT1A1. The early diagnosis of PSC is challenging. Close and dynamic detection of the patient’s biliary ductal lesions is required. In the event of relatively rare biliary complications, such as recurrent bile duct stones, attention should be paid to the presence of gene mutation. Such patients tend to have a poor prognosis and require liver transplantation at an early stage.

## Acknowledgments

We would like to extend our gratitude to Professor Xiong Ma from Renji Hospital, School of Medicine, Shanghai Jiao Tong University, for his invaluable assistance and support in diagnosing and treating this patient. We would also like to thank DeepL [https://www.deepl.com/zh/translator] for editing and reviewing this manuscript for English language.

## Author contributions

**Conceptualization:** Yijun Zhou, Jianfeng Bao, Qiaofei Jin.

**Investigation:** Yijun Zhou, Wei Shen, Yusheng Cui, Yunjiang Li, Hong Liu.

**Formal analysis:** Yusheng Cui.

**Methodology:** Yusheng Cui, Yunjiang Li, Hong Liu.

**Project administration:** Wei Shen.

**Resources:** Yunjiang Li, Jianfeng Bao.

**Supervision:** Qiaofei Jin.

**Validation:** Wei Shen, Jianfeng Bao, Qiaofei Jin.

**Visualization:** Wei Shen, Yusheng Cui, Hong Liu.

**Writing – original draft:** Yijun Zhou.

**Writing – review & editing:** Jianfeng Bao, Qiaofei Jin.
